# Antimicrobial susceptibility patterns of enterobacteriaceae isolated from HIV-infected patients in Kinshasa

**DOI:** 10.11604/pamj.2014.17.179.3788

**Published:** 2014-03-07

**Authors:** Jean-Marie Liesse Iyamba, José Mulwahali Wambale, Ntondo za Balega Takaisi-Kikuni

**Affiliations:** 1 Laboratory of Experimental and Pharmaceutical Microbiology, Faculty of Pharmaceutical Sciences, University of Kinshasa, Kinshasa, Democratic Republic of Congo

**Keywords:** HIV, enterobacteriaceae, antibiotic sensitivity, Kinshasa

## Abstract

**Introduction:**

People infected by Human Immunodeficiency Virus (HIV) are susceptible to develop severe bacterial infections. We set out to determine the frequency and the sensitivity to antibiotics of enterobaceriaceae isolated from urine and feces of HIV-infected persons.

**Methods:**

Urine and feces samples were collected from HIV-infected patients of the Centre de Traitement Ambulatoire de Kabinda (CTA/Kabinda, Kinshasa) and analyzed at the Reference National Laboratory for HIV/AIDS and Sexually Transmitted Infections. The isolated enterobacteriaceae strains were identified by conventional microbiological methods. Antibiotic sensitivity pattern was carried out by disc diffusion method.

**Results:**

The following bacteria pathogens were isolated: *Escherichia coli, Klebsiella, Enterobacter, Proteus*, and *Providencia*. Most species were sensitive to cefotaxim, ceftriaxon, and gentamicin and resistant to chloramphenicol, cotrimoxazole, tetracycline, and norfloxacin.

**Conclusion:**

The results of the present study show that the most frequently bacteria isolated were *Esherichia coli* and cefotaxim, ceftriaxon, and gentamicin were the most active antibiotics.

## Introduction

Persons with HIV are exposed to opportunistic infections caused by different microorganisms including bacteria, fungi, viruses, protozoa and helminthes [1]. These infections are consecutive to different subabnormalities of host defense against infectious agents [2, 3]. In such patients, enterobacteriaceae which are responsible for gastrointestinal and urinary tract infections may raise the incidence of HIV and the progression of HIV infection to AIDS. Bacterial opportunistic infections are treatable with broad or narrow spectrum antibiotics [4, 5]. But the control of these infections constitutes a challenge because of the emergence of multiple antibiotic resistances. Studies on susceptibility patterns of enterobacteriaceae isolated from HIV-infected persons were conducted in some African countries [6, 7], but few literature data give the current situation in the Democratic Republic of Congo. The aim of this preliminary study is first to determine the frequency of enterobaceriaceae isolated from urine and feces of HIV-infected patients in Centre de Traitement Ambulatoire de Kabinda (CTA/Kabinda), Kinshasa, and secondly, to evaluate their susceptibility patterns to antibiotics.

## Methods

Urine and feces samples from HIV infected patients used in this study were collected for diagnostic purposes in CTA/Kabinda, Kinshasa in a period of 3 months and were analyzed at the Reference National Laboratory for HIV/AIDS and Sexually Transmitted Infections. All Samples were cultured in Hektoen and Mac Conkey agars. For urine samples, colonies were counted onto Cystein Lactose Electrolyte Deficient (CLED) agar. Inoculated plates were incubated at 37°C for 24 hours.

Bacterial isolates were identified using microbiological conventional methods including Gram staining, oxydase test, indole and urease production, citrate utilization, hydrogen sulphide and gas production, and fermentation of sugars. Forty eight urine samples considered significant for urinary tract infection (bacterial account 105 CFU / ml) and 31 feces samples were studied.

Antibiograms of each isolated strains using the diffusion method on Mueller Hinton Agar were realized with the following antibiotic disks (Biomerieux, France): Ampicillin (10µg), cefotaxim (30µg), cefazolin (30µg), ceftriaxon (30µg), cefuroxime (30µg), ciprofloxacin (5µg), cotrimoxazole (1.25µg/25.75µg), chloramphenicol (30µg), gentamicin (10µg), norfloxacin and tetracycline (30µg). The results of susceptibility tests were analyzed according to the recommendations from National Committee for Clinical Laboratory Standards (NCCLS) [8].

## Results

### Distribution of enterobacteriaceae isolated from feces and urine

In the present study, we observed that the isolated enterobacterial strains from feces and urine of the HIV-infected patients were the species belonged to *Escherichia, Klebsiella, Enterobacter*, and *Proteus-Providencia*. These results showed further that Escherichia coli was the most frequently isolated pathogen as well in feces with 27 strains (87%) as in urine with 30 trains (62.5%), followed by strains of *Klebsiella pneumonia* and *Enterobacter gergoviae* (Table 1, Table 2).

**Table 1:** Distribution of enterobacteriaceae isolated from feces samples

Bacteria	Frequency	Percentage (%)
*E. coli*	27	87.0
*Klebsiella pneumoniae*	2	6.5
*Enterobacter agglomerans*	1	3.25
*Proteus vulgaris*	1	3.25
**Total**	**31**	**100**

**Table 2:** Distribution of enterobacteriaceae isolated from urine samples

Bacteria	Frequency	Percentage (%)
***E. coli***	**30**	**62.5**
*Klebsiella*	**9**	**18.75**
*Klebsiella pneumoniae*	6	12.5
*Klebsiella ozaenae*	2	4.125
*Klebsiella oxytoca*	1	2.125
***Enterobacter***	**6**	**12.5**
*Enterobacter agglomerans*	1	2.1
*Enterobacter gergoviae*	4	8.3
*Enterobacter aerogenes*	1	2.1
***Proteus***	**3**	**6.25**
*Proteus mirabilis*	2	4.125
*Providencia rettgeri*	1	2.125
**Total**	**48**	**100**

### Antibiotic sensitivity testing

The results of antibiotic susceptibility testing of the enterobacterial strains isolated from feces showed that the majority (88.8%) of *Escherichia coli* and 2 *Klebsiella pneumoniae* strains were highly susceptible to cefotaxim and ceftriaxon, whereas 20 (74.1%) *Escherichia coli* strains and the other strains tested, except those of *Enterobacter agglomerans*, were susceptible to gentamicin. *Enterobacter agglomerans* and *Proteus vulgaris* were resistant to the majority of antibiotics. Fourteen *Escherichia coli* strains (51.8%) were sensitive to ciprofloxacin and only 7 (25.5%) sensitive to norfloxacin. The lowest frequency of sensitivity for *Escherichia coli* was observed with cotrimoxazole and tetracycline 1(3.7%), followed by amoxicillin and chloramphenicol 2 (7.4%) (Table 3).

**Table 3:** Susceptibility patterns to antibiotics of enterobacteriaceae isolated from fece to antibiotics

	Bacteria							
**Antibiotics**	*Escherichia coli*		*Klebsiella pneumoniae*		*Enterobacteragglomerans*		*Proteus vulgaris*	
	**S**	**R**	**S**	**R**	**S**	**R**	**S**	**R**
Ampicillin	2(7.4%)	25(92.6%)	0(0%)	2(100%)	0 (0%)	1(100%)	0(0%)	1(100%)
Cefotaxim	24(88.8%)	3(11. 2%)	2(100%)	0(0%)	0(0%)	1(100%)	1(100%)	0(0%)
Cefazolin	5(18.5%)	22(81.5%)	0(0%)	2(100%)	0(0%)	1(100%)	0(0%)	1(100%)
Ceftriaxon	24(88.8%)	3(11.2%)	2(100%)	0(0%)	0(0%)	1(100%)	1(100%)	0(0%)
Cefuroxin	7(25.9%)	20(74.1%)	0(0%)	2(100%)	0(0%)	1(100%)	0(0%)	1(100%)
Ciprofloxacin	14(51.8%)	13(48.2%)	1(50%)	1(50%)	1(100%)	0(0%)	0(0%)	1(100%)
Cotrimoxazole	1(3.7%)	26(96.3%)	0(0%)	2(100%)	0(0%)	1(100%)	0(0%)	1(100%)
Chloramphenicol	2(7.4%)	25(92.6%)	0(0%)	2(100%)	0(0%)	1(100%)	0(0%)	1(100%)
Gentamicin	20(74.1%)	7(25.9%)	2(100%)	0(0%)	1(100%)	0(0%)	0(0%)	1(100%)
Norfloxacin	7(25.5%)	20(74.1%)	1(50%)	1(50%)	0(0%)	1(100%)	0(0%)	1(100%)
Tetracycline	1(3.7%)	26(96.3%)	0(0%)	2(100%)	0(0%)	1(100%)	0(0%)	1(100%)
**Strains tested**	**27**		**2**		**1**		**1**	

Data about antibiotic susceptibility testing of the enterobacterial strains isolated from urine showed that *Escherichia coli* strains were highly sensitive to cefotaxim 26 (89.6%) and ceftriaxon 27 (93.1%), whereas they were highly resistant to tetracycline 29 (100%), ampicillin and cotrimoxazole 27 (93.1%), followed by chloramphenicol 26 (89.6%), cefuroxim 25 (86.2%), and cefazolin 23 (79.3%). All *Enterobacter* species and 21 (72.4%) *Escherichia coli* strains were sensitive to gentamicin. *Enterobacter, Klebsiella* and *Proteus-Providencia* strains were generally sensitive to ciprofloxacin, but the majority of *Escherichia coli* strains were resistant to the 2 fluoroquinolones tested in this study (Table 4, Table 5).

**Table 4:** Susceptibility patterns to antibiotics of enterobacteriaceae isolated from urine

	Bacteria							
**Antibiotics**	*Escherichia coli*		*Klebsiella pneumoniae*		*Klebsiella ozaenae*		*Klebsiella oxytoca*	
	**S**	**R**	**S**	**R**	**S**	**R**	**S**	**R**
Ampicillin	2 (6.9%)	27(93.1%)	0(0%)	6(100%)	1(50%)	1(50%)	0(0%)	1(100%)
Cefotaxim	26(89.7%)	3(10.3%)	5(83.3%)	1(16.7%)	2(100%)	0(0%)	1(100%)	0(0%)
Cefazolin	6(20.7%)	23(79.3%)	4(66.7%)	2(33.3%)	0(0%)	2(100%)	0(0%)	1(100%)
Ceftriaxon	27(93.1%)	2(6.9%)	5(83.3%)	1(16.7%)	2(100%)	0(0%)	0(0%)	1(100%)
Cefuroxin	4(13.8%)	25(86.2%)	2(33.3%)	4(66.7%)	1(50%)	1(50%)	0(0%)	1(100%)
Ciprofloxacin	14(48.3%)	15((51.7%)	4(66.7%)	2(33.3%)	0(0%)	2(100%)	1(100%)	0(0%)
Cotrimoxazole	2(6.9%)	27(93.1%)	1(16.7%)	5(83.3%)	0(0%)	2(100%)	0(0%)	1(100%)
Chloramphenicol	3(10.3%)	26(89.7%)	1(16.7%)	5(83.3%)	0(0%)	2(100%)	0(0%)	1(100%)
Gentamicin	21(72.4%)	8(27.6%)	4(66.7%)	2(33.3%)	1(50%)	1(50%)	1(100%)	0(0%)
Norfloxacin	10(34.5%)	19(69.5%)	3(50%)	3(50%)	0(0%)	2(100%)	0(0%)	1(100%)
Tetracycline	0(0%)	29 (100%)	1(16 .7%)	5(83.3%)	0(0%)	2(100%)	0(0%)	1(100%)
**Strains tested**	**29**		**6**		**2**		**1**	

## Discussion

The analysis of urine and feces samples from HIV-infected patients has allowed the identification of five enterobacteriacea major groups: *Escherichia, Enterobacter, Klebsiella, Proteus* and *Providencia*. As shown in Table 1 and Table 2, *E. coli* was the predominant pathogen isolated from urine and feces.

Regarding gastrointestinal infections, *Escherichia coli* is known to be the most important enteric pathogen encountered in persons who are not HIV infected [9, 10]. It has been also demonstrated that this pathogen was involved in diarrhea in HIV pediatric patients [11, 12]. *Escherichia coli* remains the predominant Gram negative uropathogen isolated in acute community uncomplicated infections, followed by *Klebsiella, Enterobacter* and *Proteus* species [13]. Results obtained in this study were consistent with those reported by other authors [14, 15].

The results of antimicrobial susceptibility tests revealed that only 3 antibiotics (cefotaxim, ceftriaxon and gentamicin) were active against the majority of the studied strains, especially *Escherichia coli* and *Klebsiella* from HIV-infected patients. Report from Bamako in Mali [15] demonstrated that these two species were sensitive to cefotaxim and gentamicin. *Proteus* and *Providencia* strains had variable sensitivity to gentamicin. About 50% of the isolated enterobacterial strains were sensitivity to ciprofloxacin. However, all strains were generally resistant to cefazoline and cefuroxim, indicating probably that these strains produce extend spectrum betalactamase (ESBL).

The majority of strains were highly resistant to ampicillin, chloramphenicol, cotrimoxazole, tetracycline and norfloxacin. A study from Jamaica [16] reported that *Escherichia coli* strains from urine of HIV-infected children were resistant to cotrimoxazole. This antibiotic is used to reduce mortality and morbidity in HIV-infected persons in Africa. However, a study from Kenya [17] showed that the prevalence of cotrimoxazole resistance increased significantly from 78% to 98% among persons taking daily cotrimoxazole. Another study from Tanzania [18] reported that fecal *Escherichia coli* resistant to cotrimoxazole was resistant to ampicillin, chloramphenicol, ciprofloxacin and nalidixic acid. As demonstrated in all these studies, cotrimoxazole and the other antibiotics tested became poor choice for an empiric treatment of urinary and gastrointestinal tract infections in HIV-infected patients. The antibiotic resistance of enterobacteriaceae presents now a worldwide worrying evolution with an increasing ESBL production. Because of limited data regarding the antimicrobial resistance of enterobacteriaceae-producing ESBL in HIV-infected patients from DRC, we are currently conducting further investigations in HIV-uninfected and HIV-infected persons.

## Conclusion

The present study demonstrated that *Escherichia coli* was the most frequently isolated bacteria, and cefotaxim, ceftriaxon and gentamicin the most active antibiotics which should be recommended in the treatment of enterobacteriaceae infections in HIV-infected patients. The data obtained showed high resistance of enterobacteriaceae strains to the majority of antibiotics screened. Therefore, a continuous antibiotic surveillance may be necessary in DRC where antibiotics are usually used without prescriptions.
